# Establishment of a juvenile mouse asthma model induced by postnatal hyperoxia exposure combined with early OVA sensitization

**DOI:** 10.1016/j.heliyon.2023.e23291

**Published:** 2023-12-03

**Authors:** Jingyan Li, Tianping Bao, Linxia Cao, Mengmeng Ma, Bingrui Yu, Yuan Zhang, Rong Wu, Haiyan Zhu, Zhaofang Tian

**Affiliations:** a Department of Neonatology, The Affiliated Huaian No.1 People's Hospital of Nanjing Medical University, Huai 'an, Jiangsu, 223300, China; b Neonatal Medical Center, Huaian Maternity and Child Healthcare Hospital, Anhui Medical University, Huai'an, Jiangsu, 223002, China

**Keywords:** Asthma, Hyperoxia, Newborn, Mice, Model

## Abstract

**Objective:**

To establish a juvenile mouse asthma model by postnatal hyperoxia exposure combined with early ovalbumin (OVA) sensitization.

**Methods:**

Female C57BL/6J newborn mice were exposed to hyperoxia (95 % O_2_) from postnatal day-1 (PND1) to PND7; intraperitoneally injected with OVA suspension on PND21, PND28; and stimulated by nebulized inhalation of 1 % OVA from PND36 to PND42. Within 48 h of the last challenge, we observed their activity performance and evaluated airway responsiveness (AHR). All mice were executed at PND44. Female (n = 32) were divided into four groups as follows: room air（RA）+phosphate-buffered saline (PBS) group; O_2_ (hyperoxia, 95 % O_2_) + PBS group; RA + OVA group; O_2_+OVA group. We obtained the serum, bronchoalveolar lavage fluid (BALF), and lung tissues. The Wright-Giemsa staining was performed for leukocyte classification in BALF and HE staining for pathological examination. The levels of IL-2, IL-5, IL-13, IL-17A and IL-10 in BALF and tIgE and sIgE in serum were detected by ELISA.

**Results:**

Compared with OVA sensitization or hyperoxia exposure alone, the mice in the model group (O_2_+OVA) showed asthma-like symptoms and increased airway hyperreactivity,The levels of IL-5,IL-13 IL-17A were increased in BLAF,and total leukocyte and eosinophil counts were also significant increasesed. The levels of tIgE and sIgE in serum were increased.

**Conclusion:**

Postnatal hyperoxia exposure combined with early OVA sensitization might establish a juvenile mouse asthma model.

## 1 Introduction

Asthma is an allergic inflammatory disease characterized by airway hyperresponsiveness and affects about 300 million people worldwide. It usually begins in childhood (especially before the age of 5), and approximately 40 % of children and adolescents worldwide suffer from asthma to varying degrees [1]. In children, persistent asthma leads to insufficient lung function development, increases the risk of chronic obstructive pulmonary disease (COPD), persistent airflow obstruction in adulthood, and reduces the growth rate of children. In addition, a severe acute attack can lead to sudden death [2]. Hence, it is essential to understand the pathophysiological mechanisms of asthma and develop effective treatments, for which animal models are highly valuable.

The establishment of animal models of asthma is the basis for studying the pathogenesis and development of asthma. There are many kinds of animal asthma models and various research methods. A wide variety of animal species, such as mice, horses, rats, dogs, sheep, and monkeys, have been used for studies of airway inflammation in asthma [3]. Of these, mouse models are the most common [4]. However, these models were established in adult mice (6–8 weeks old), and whether they can represent the characteristics of childhood asthma is not yet determined. Therefore, establishing a juvenile asthma model will aid in a better understanding of childhood asthma.

Supplemental O_2_ (hyperoxia) is necessary for preterm infant survival; however, early oxygen exposure clearly associates with airway hyperreactivity later in life [5]. Hyperoxia in neonatal mice increases airway reactivity, airway remodeling associated with inflammation, and a significant increase in lymphocytes, which lead to a continuous inflammatory response in the lungs of adult mice [6]. Cheon et al. reported that the expression of type 2 cytokine genes IL-13 and IL-5 increased after hyperoxia exposure in the lungs of 4-week-old mice, indicating that hyperoxia injury leads to type 2 inflammation [7]. Our previous research in a 6-week-old mouse model of OVA-induced asthma found that early postnatal exposure to 95 % O_2_ for 7 days can aggravate the airway inflammation, which was accompanied by obvious airway structural remodeling, and aggravated shift of th1/th2 [8]. But by the end of modeling, the mice were in adulthood and might have difficulty characterizing asthma in children under 14 years of age.

This study is on the basis of previous studies [30], we will advance the OVA induction to 3 weeks after the birth of mice, modeling at the end of the mouse 6 weeks of age, so the entire modeling procedure was performed at the juvenile stage.

## 2 Materials & methods

### 2.1 Animal experimental procedure

**Fig. 1:**
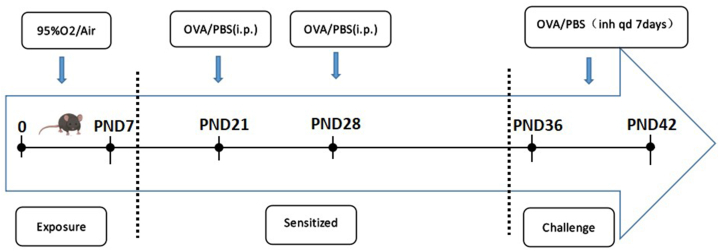
Experimental design and timelines.

Newborn C57BL/6J mice (SPF grade) were exposed to either room air or 95 % oxygen until postnatal day 7. Hyperoxic dams were exchanged with normoxic dams every 48 h to avoid oxygen toxicity. Then, the mice intraperitoneal (i.p.) injectionwith 100 μL sensitization solution [OVA 1 mg/ml + Al(OH)3 1 mg/ml] or equal amounts of PBS at PND21, PND28. After this, the mice were then challenged on 7 consecutive days (PND36- PND42)with 1 % OVA or equal amounts of PBS aerosol for 30 min. Flow chart of mouse model establishment is shown in Fig. 1. The experimental groups were as follows: room air（RA）+phosphate-buffered saline (PBS) group; O2 (hyperoxia, 95 % O2) + PBS group; RA + OVA group; O2+OVA group（n = 8). Within 48 h of the last challenge, we measured the airway reactivity of mice with a lung function instrument and observed their activity performance. All mice were executed by asphyxiation in CO2 on PND44. The mice used in this experiment were all female neonates within 24 h of birth. C57BL/6J mice (SPF grade) were obtained from The Medical Animal Experiment Center of Nanjing Medical University. This experiment was approved by the ethics committee of the Affiliated Huaian No. 1 People's Hospital of Nanjing Medical University.（DW-P-2021-00201）

### 2.2 Lung mechanics

Within 48 h of the last challenge, we determined the airway reactivity of mice by the invasive method of mouse lung function apparatus. After anesthetizing the mice with 1 % pentobarbital sodium 50 mg/kg(i.p.), we performed endotracheal intubation. Then the ventilator was connected to determine the change in airway resistance in mice. Methacholine was added to the instrument to observe the changes in airway reactivity when mice were exposed to 0, 6.25, 12.5, 25, and 50 mg/ml of methacholine(inh). Airway resistance (cm H2O.s/mL) was measured using the flexiVent system (SCIREQ, Montreal, Quebec, Canada).

### 2.3 Serum IgE level

A small volume of blood was collected from the heart of the mice for measuring total IgE(tIgE) and ST-specific IgE (sIgE) levels in sera. The ELISA kit was purchased from NeoBioscien Company, and the experimental steps were strictly in accordance with the kit instructions.

### 2.4 Leukocyte classification and cytokine levels in BALF

After assessment of methacholine responsiveness, mice were sacrificed and BALF was collected by lavage of the trachea, lungs, and bronchi. For this, 1 ml sterile saline in a 1 ml syringe pump was slowly pushed into the physiological saline. Leukocytes were classified by Wright-Giemsa staining. The levels of cytokines, such as IL-2, IL-5, IL-13, IL-17A, and IL-10, were detected using ELISA kits purchased from NeoBioscien Company according to the manufacturer's instructions.

### 2.5 Histopathological examination

Both lungs and trachea were removed, visualized with the naked eye, and then rinsed with PBS. The upper lobe of the right lung was fixed in 4 % paraformaldehyde, paraffin-embedded, and cut into serial sections of 4 μm thickness for histopathological examination. Changes in airway thickness and columnar epithelial hyperplasia were observed under light with H&E staining. The ratio of airway wall thickness to total airway area was calculated, and images were captured. The pathology of each group was graded and scored [9].

### 2.6 Statistical analysis

The SPSS 21.0 statistical software was used for the statistical analysis of the data. Data were represented as mean ± standard deviation (SD). Statistical significance was determined to be P < 0.05 (*), P < 0.01 (**), or not significant if P > 0.05.

## 3 Results

### 3.1 Performance of mice

**Fig. 2:**
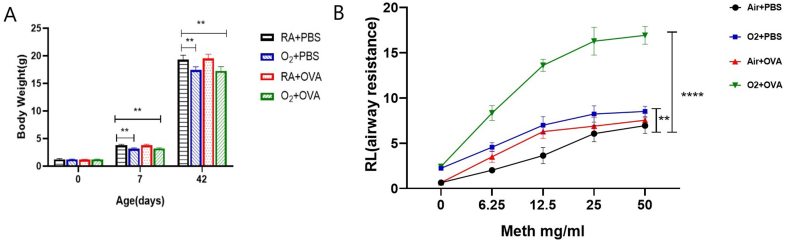
Performance of the mice in four groups. (A) Weight of mice at different time points (n = 8). (B) Pulmonary function following methacholine injection (n = 8). Data reported as means ± S. Significant results are shown as *P ≤ 0.05, **P ≤ 0.01.

The mice after early hyperoxia exposure had smaller body sizes (Fig. 2A). In addition, mice in the O_2_ + OVA group developed asthma-like symptoms, such as scratching around the nose, curled body, and difficult breathing after OVA excitation.

### 3.2 Airway resistance

At baseline, the airway resistance was slightly higher in the O_2_ + PBS and O_2_ + OVA groups than RA + PBS and RA + OVA groups. Additionally, airway resistance of RA + OVA and O_2_+OVA groups showed an upward trend with an increase in Mch dose. When the stimulation concentration of Mch increased to 12.5 mg/ml, the airway resistance of the O_2_ + OVA group increased significantly (P < 0.01) compared with the RA + PBS group(Fig. 2B).

### 3.3 Pathological examination

**Fig. 3:**
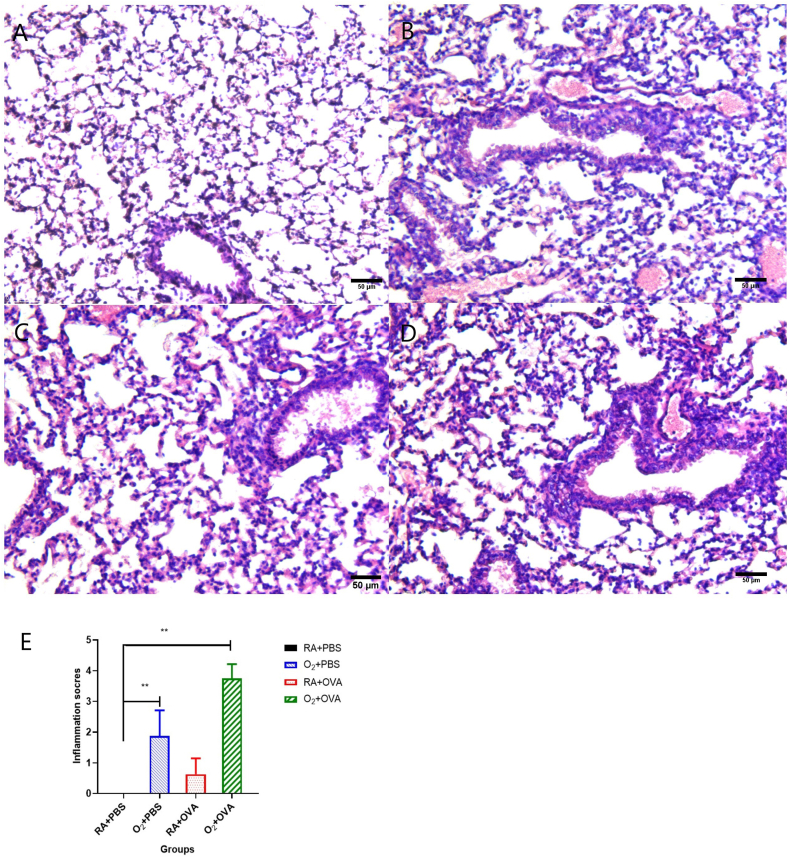
Bronchial pathological changes.(H&E staining × 400, scale bar = 25 μm) (A) RA + PBS group; (B)O_2_ + PBS group; (C) RA + OVA group; (D) O_2_ + OVA group (E) Lung inflammation scores in different groups(**P ≤ 0.01).

The lung sections stained with H&E revealed inflammation in the alveolar and small airways. In the O2 + PBS group, the airway wall was marginally thicker than in the RA + PBS group (Fig. 3A and B). The RA + OVA group showed hypertrophy of the columnar epithelium of the airways, but there was no discernible increase in the thickness of the airways. The O2 + OVA group had a much thicker airway wall along with considerable luminal stenosis and inflammatory cell infiltration (Fig. 3C,D,E).

### 3.4 Cell classification in BALF

**Fig. 4:**
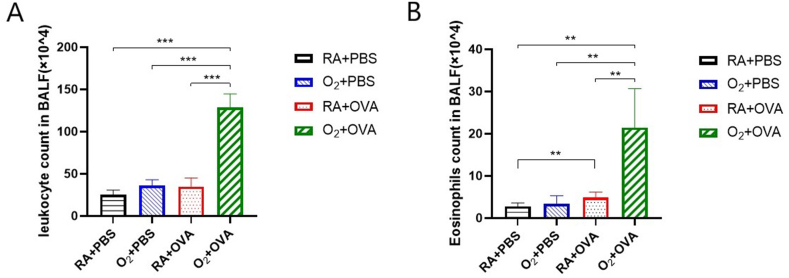
Airway inflammation in BALF.（n = 8） (A) Leukocyte counts in BALF. (B) Eosinophil counts in BALF. **P ≤ 0.01,***P < 0.001.

As shown in Fig. 4A and B, the O2 + OVA group mice had significantly higher total leukocyte and eosinophil counts in BALF compared to the other three groups.(P < 0.05).

### 3.5 BALF cytokines levels

**Fig. 5:**
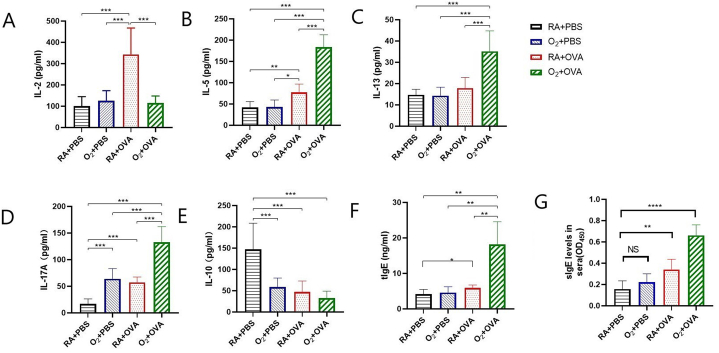
BALF cytokines and serum IgE (n = 8) (A) IL-2. (B) IL-5. (C) IL-13. (D) IL-17A. (E) IL-10 levels in BALF. (F) tIgE level in serum.(G) sIgE level in serum *P＜0.05，**P < 0.01 ***P < 0.001.

3.5.1 Th1 cytokine production in BALF: Compared to the mice in the RA + OVA group, the O2 + OVA group's BALF had considerably lower levels of IL-2 (P < 0.05). The O2 + OVA group did not significantly vary from the other two groups (P > 0.05). (Fig. 5A).

3.5.2 Th2 cytokine production in BALF:In the BALF of the O2 + OVA group, there was a significant rise in the expression of type 2 cytokines, IL-5 and IL-13. When compared to the other three groups, the mice in the O2 + OVA group exhibited noticeably greater IL-5 levels in BALF (P < 0.05). Mice in the Air + OVA group had significantly greater levels of IL-5 in their BALF compared to the O2 + PBS and Air + PBS groups (P < 0.05). Fig. 5B.When compared to the other three groups, the mice in the O2 + OVA group exhibited noticeably greater IL-13 levels in BALF (P < 0.05). The remaining three groups did not differ significantly from one another (P > 0.05) (Fig. 5C).

3.5.3 Th17/Treg response in BALF:In comparison to the other three groups, the mice in the O2 + OVA group had significantly greater levels of IL-17A in their BALF (P < 0.05) (Fig. 5D). In comparison to the RA + PBS group, the BALF of mice in the O2 + PBS, RA + OVA, and O2 + OVA groups had significantly lower levels of IL-10 (P < 0.05). The O2 + PBS, Air + OVA, and O2 + OVA groups did not, however, differ significantly (P > 0.05) (Fig. 5E).3.6 Serum IgE level:

3.5.4 Levels of tIgE and sIgE in sera were significantly higher in mice of the O_2_ + OVA group compared with those of the other three groups (P < 0.01)，and RA + OVA groups were higher compared with that in the RA + PBS group(P < 0.05), as shown in Fig. 5F–G.

## 4 Discussion

Hyperoxia exposure during the neonatal period promotes the development of diseases such as bronchopulmonary dysplasia (BPD) and bronchial asthma. BPD is associated with an increased risk of wheezing episodes and asthma symptoms in children [10]. Besides, studies have shown that preterm and very low birth weight young people have lower rates of specific response than young people born at term [11]. This suggested that exposure to hyperxic in preterm infants may increase the risk of anaphylactic immune reactions from allergen exposure [12,13].

Early postpartum hyperoxia exposure can aggravate airway inflammation, accompanied by significant airway structural remodeling and airway hyperresponsiveness. In this study, mice in the model group (O_2_ + OVA) showed obvious asthma-like symptoms. Pulmonary function showed a significant increase in AHR. The pathological sections of this group of mice exhibited significant thickening of the airway wall, and significant luminal stenosis and airway columnar epithelial hyperplasia. Early hyperoxia exposure can produce reactive oxygen species (ROS) [5]. ROS can damage to the airway epithelium and disrupt cellular function, eventually leading to an increase in airway smooth muscle, increased extracellular interstitial deposition around the airway, and cellular senescence, affecting the integrity of the airway epithelial barrier and producing airway remodeling [14,15]. Previous studies have shown that hyperoxia exposure leads to neonatal lung injury and airway hyperreactivity by disrupting the nitric oxide (NO) and cyclic guanosine phosphate (cGMP) signaling and increasing arginase activity [16].

The complex immune response of asthma can lead to the loss of Th1 cell expression and the increase of Th2 cell expression, resulting in Th1/Th2 imbalance [17]. After hyperoxia and sensitization, the destruction of pro-inflammatory immune response and lung development in early mice is exaggerated. Pulmonary homeostasis and type 2 cytokine environment are disturbed at the critical stage of lung development, leading to Th1/Th2 cell imbalance [[18], [19], [20]]. The results of this experiment showed that the airway height of the model group was increased, the total number of white blood cells and eosinophils in BALF were significantly increased, and the levels of IL-5 and IL-13 were significantly increased, but there was no significant difference in the level of IL-2, which was consistent with the Th1/Th2 imbalance and the characteristics of Th2 dominance. Early life hyperoxia exposure may enhance ILC2 function through increased IL-33 expression [21]. ILC2s were a major source of IL-5 or IL-13 [22]. And ILC2s can promote antigen uptake and dendritic cell migration to the lung-draining lymph nodes, thereby facilitating the induction of Th2 responses against allergens. In addition, the level of IL-2 in RA + OVA group was significantly higher than that in control group, indicating that OVA sensitization in air environment may lead to immune tolerance in mice.

Recent studies found that Th17/Treg imbalance is an important mechanism of asthma. IL-17 recruits granulocytes and increases the Th2-mediated eosinophil inflammatory response, which is related to the severity of asthma [23]. Tregs mainly secrete TGF-β, IL-10, and other anti-inflammatory factors, inhibit the activation and proliferation of effector T cells, inhibit the Th2 and Th17-mediated inflammatory response, and prevent airway inflammation and bronchial hyperresponsiveness [24]. Some studies believe that hyperoxia can significantly change the microenvironment of inflammatory sites, thereby inducing IL-17 expression in lung tissues. This is consistent with the known hyperoxia effect, including the induction of MPO and IFN- γ, IL-1A, IL-4, IL-5, IL-6, IL-13, IL-17, TNFα, VEGF, CCL2, and TGF-β [25]. Some Studies have shown that hyperoxia can lead to an increase in protective IL-10 during acute injury. However, the 2HIT model of hyperoxia showed that mRNA expression levels of IL-4, IL-10, and IL-13 cytokines begin to decrease during the recovery phase at the end of hyperoxia [26]. This study found that IL-17A levels increased significantly, IL-10 levels decreased, and there was a significant imbalance in the Th17/Treg ratio in BALF of mice belonging to the O2 + OVA group.

However, there are conflicting reports on whether neonatal hyperoxia exposure can enhance allergic reactions to OVA. Previous research has shown that exposing mice to hyperoxia soon after birth and then making them sensitive to OVA as adults did not enhance their response to OVA [27]. We think that it is caused by different excitation time points. There is a “time window” for the impact of allergen exposure on asthma [28]. During the neonatal period of alveolar formation (alveolarization)，which lasts up to three years in humans, there is a rapid increase in type 2 immune cells that are involved in the type 2 immune response. This is called a type 2 immunological bias, and it is often linked to allergies [29]. Leading to increased susceptibility to immune-mediated diseases such as asthma.

## 5 Conclusion

In all, we established a juvenile mouse asthma model by postnatal hyperoxia exposure combined with early OVA sensitization. Using this model, we studied the characteristic symptoms of asthma, such as airway inflammation. However, we need to further evaluate this asthma model to confirm its consistency with clinical pediatric asthma.

## Ethics statement

The animal study was reviewed and approved by the Ethics Committee of the Affiliated Huaian No. 1 People's Hospital of Nanjing Medical University (DW-P-2021-002-01).

## Funding

This study was financially supported by Key scientific research project of Jiangsu Provincial Health Commission (ZDB2020005) and 10.13039/501100007289Nanjing Medical University Science and Technology Development Fund (NJUB20210139).

## Availability of data and materials

The dataset generated or analyzed during this study can be made available to interested researchersby the authors of his article upon reasonable request.

## CRediT authorship contribution statement

**Jingyan Li:** Conceptualization, Writing – original draft. **Tianping Bao:** Conceptualization, Data curation, Formal analysis. **Linxia Cao:** Investigation, Methodology. **Mengmeng Ma:** Investigation, Methodology. **Bingrui Yu:** Investigation, Methodology. **Yuan Zhang:** Data curation, Resources. **Rong Wu:** Investigation, Methodology. **Haiyan Zhu:** Writing – review & editing. **Zhaofang Tian:** Conceptualization, Writing – original draft, Writing – review & editing.

## Declaration of competing interest

The authors declare that they have no known competing financial interests or personal relationships that could have appeared to influence the work reported in this paper.
